# O045. Cluster headache improvement during Ketogenic Diet

**DOI:** 10.1186/1129-2377-16-S1-A99

**Published:** 2015-09-28

**Authors:** Cherubino Di Lorenzo, Gianluca Coppola, Giulio Sirianni, Paolo Rossi, Francesco Pierelli

**Affiliations:** 1 Don Carlo Gnocchi Onlus Foundation, Milan, Italy; 2 G.B. Bietti Foundation-IRCCS, Department of Neurophysiology of Vision and Neuro-ophthalmology, Rome, Italy; 3 Wellness and Dietary Medicine, Krom Genetics Institute, Rome, Italy; 4 Headache Clinic, INI, Grottaferrata (RM), Italy; 5 University Consortium for Adaptive Disorders and Head pain (UCADH), Pavia, Italy; 6 Department of Medico-Surgical Sciences and Biotechnologies, Sapienza University of Rome, Latina, Italy; 7 IRCCS - Neuromed, Pozzilli (IS), Italy

## Introduction

Ketogenic diet (KD) is a valid treatment for drug-resistant epilepsy, recently proposed as effective also in migraine. Hitherto, no data are available about KD effects on cluster headache (CH), a severe form of primary headache, characterized (similarly to migraine) by a trigemino-vascular activation. Prophylaxis drug-resistant patients have a great need for care and could seek help in invasive treatments and/or alternative drugs, such as illegal substances. Here we performed a prospective observational study of the potential beneficial ketogenesis-induced effects on CH, studying a group of consecutive drug-resistant CH patients.

## Methods

We recruited 12 CH drug-resistant patients (7 chronic, CCH) that accepted to undergo a 3-month trial with KD in order to try to treat their headache. Patients received a ketogenic “modified Atkins diet” or “Classic Diet” characterized by a 3:1 ratio (75% fat, 25% non-fat macronutrients). During ketogenesis, patients underwent medical supervision and standard laboratory blood tests. At the end of the KD, patients were free to decide whether to prolong ketogenesis or revert to their standard diet (SD).

## Results

Out of 5 episodic CH (ECH) patients, all fully responded to the diet at the end of the first month (three became headache free in a couple of weeks, the other two reported to have had “shadows” and mild attacks up to the end of first month). All of them, at the end of KD period reverted to a standard diet and, since out of active phase of disease, have not had bouts as yet. Out of 7 CCH patients, six reported a progressive reduction of bouts in terms of number and intensity (three during the first 4-week period, one during the second 4-week period, one during the third 4-week period). At the end of the 3-month KD period, four patients reported not yet having had attacks, one reported only 'shadows’ and one reported having 2-3 attacks per week. One of patients that responded in the first month of diet decided to revert to SD and CH recurred after 7 weeks.

## Discussion

Drug-resistant CH is one of the greatest challenges in headache medicine, and new therapeutic options are welcome. Our observation suggests that ketogenesis can also help CH patients other than migraineurs, maybe by modulation of cortical excitability, or by dampening neural-inflammation. It is interesting to note that both CH and migraine are headache forms that involve the trigeminovascular system, maybe the real target of ketogenesis in headache patients.

Written informed consent to publication was obtained from the patient(s).

